# Budd-Chiari syndrome in a 25-year-old woman with Behçet's disease: a case report and review of the literature

**DOI:** 10.1186/1752-1947-5-52

**Published:** 2011-02-07

**Authors:** Daniela T Carvalho, Fernando T Oikawa, Nilce M Matsuda, Paulo RB Évora, Alice T Yamada

**Affiliations:** 1 Hospital Municipal Dr. Fernando Mauro Pires da Rocha, São Paulo, Brazil; 2 Departamento de Cirurgia e Anatomia, Faculdade de Medicina de Ribeirão Preto, Universidade de São Paulo, Ribeirão Preto, Brazil; 3 Instituto do Coração, Faculdade de Medicina, Universidade de São Paulo, São Paulo, Brazil

## Abstract

**Introduction:**

The risk that patients with Behçet's disease will develop thrombotic complications has been previously described. Although it is distributed worldwide, Behçet's disease is rare in the Americas and Europe. Even though the pathogenic mechanisms of vascular complications of Budd-Chiari syndrome in patients with Behçet's disease are unknown, severe vascular complications of Budd-Chiari syndrome associated with Behçet's disease seem to affect mainly young men.

**Case presentation:**

We report a case of Budd-Chiari syndrome, a severe vascular complication that developed in a 25-year-old Afro-Brazilian woman with Behçet's disease.

**Conclusion:**

Severe vascular complications of Budd-Chiari syndrome in patients with Behçet's disease are much more common in young adult male patients; we present a rare case of Budd-Chiari syndrome in a young Afro-Brazilian woman with Behçet's disease.

## Introduction

The risk that young male patients with Behçet's disease will develop thrombotic complications has been previously described [1-3]. Although it has a worldwide distribution, Behçet's disease is rare in the Americas and Europe. The purpose of this article is to present an unusual case of Budd-Chiari syndrome in a young Afro-Brazilian woman with Behçet's disease and review the literature of Budd-Chiari syndrome in association with Behçet's disease.

Budd-Chiari syndrome is caused by blood clots that completely or partially block the large veins that carry blood from the liver (hepatic veins) into the inferior vena cava [4,5]. Some people have no overt symptoms, but others experience fatigue, abdominal pain, nausea, jaundice, an enlarged liver and spleen, edema in the legs, ascites, and sometimes rupture and bleeding in the varicose veins of the esophagus. Symptoms usually develop gradually over weeks or months, and Doppler ultrasonography can detect narrowed or blocked veins [4,5]. Budd-Chiari syndrome is suspected when the patient has an enlarged liver, ascites, liver failure or cirrhosis when there is no obvious cause, even after testing [4,5].

Even though the pathophysiology is unknown, the diagnosis of Budd-Chiari syndrome in patients with Behçet's disease is responsible for 3% of cases of Budd-Chiari syndrome, and the risk that patients with Behçet's disease will develop thrombotic complications is several times higher [1-3].

Behçet's disease is a multisystem disorder presenting with recurrent oral or genital ulcerations and chronic relapsing uveitis that may cause blindness and neurologic impairments; the diagnosis is clinical because there are no specific evidence, pathognomonic symptoms or specific laboratory findings [6-8].

According to the international criteria, the diagnosis of Behçet's disease requires the presence of recurrent oral ulceration in the absence of other clinical explanations along with two of the following: recurrent genital ulceration, eye lesions, skin lesions or a positive skin pathergy test [6-8]. Pathergy is the term used to describe hyper-reactivity of the skin that occurs in response to minimal trauma. Although it has a worldwide distribution, Behçet's disease is rare in the Americas and Europe and is more prevalent in Turkey and the Middle and Far East, affecting mainly young adults, with men having more severe vascular complication with this disease [1,9-11].

## Case presentation

A 25-year-old Afro-Brazilian woman was hospitalized in a public hospital with the following complaints: ascites, dyspnea after exercise, and the development of veins and edema in the abdominal wall and swelling in the legs. Five years earlier, she had developed asymmetric recurrent migratory arthritis in her wrists and ankles, moderate and intermittent fever, recurrent painful ulcers and lesions in the oral cavity and vagina, and painful transient erythema nodosum on her forearm and legs. She reported recurrent erysipelas, light smoking and moderate alcoholism. She denied abortion, use of oral contraceptives and a pathological family history.

Physical examination showed that the patient had mild dyspnea, jaundice, pale skin, absence of fever and jugular turgescence, adenopathy, acneiform eruptions on the face and trunk, reduced vesicular murmur at right lung base, ascites with varicose veins in the abdomen near the skin surface, an enlarged and tender liver and edema of legs (++/4). She developed a rapid increase in the abdominal volume, abdominal pain and dyspnea after exercise and onset of jugular turgescence.

**Figure 1:**
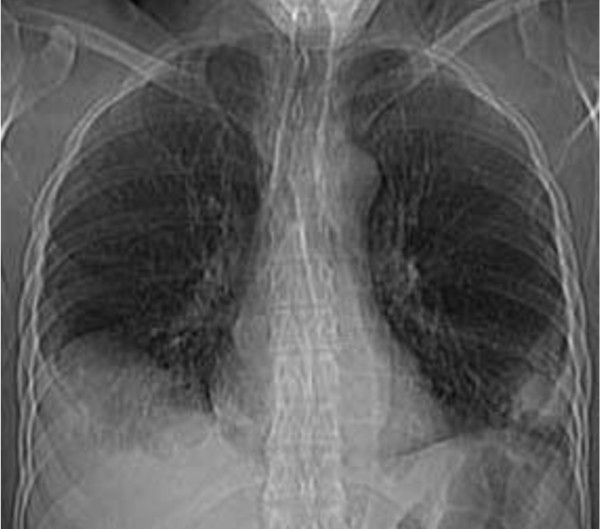
**Chest computed tomography (CT) examination of the patient**. This chest CT showed pleural effusion on the right pulmonary base.

Laboratory tests detected hypochromic and microcytic anemia; nonreactive viral hepatitis serology; nonreactive HIV and syphilis infection serology; negative autoantibodies; undetected rheumatoid factor and serum complement; normal levels of protein C, S, and antithrombin II; high hemosedimentation velocity and C-reactive protein; serum ascites albumin gradient greater than 1.1; normal indirect binocular ophthalmoscopy; and a positive skin pathergy test. A vaginal histopathology of the lesion showed nonspecific chronic inflammatory process. The chest radiograph and computed tomography examination of the patient showed pleural effusion on the right pulmonary base (Figure 1). An echocardiogram showed no pulmonary hypertension but mild systolic deficit by diffuse hypokinesia of the left ventricle, pulmonary artery pressure of 25 mm Hg, ejection fraction of 40% and mild pericardial effusion. Doppler ultrasound examination of suprahepatic and cava veins showed absence of flow in the left suprahepatic vein.

Symptomatic treatment was established and added antibiotic therapy and use of angiotensin-converting enzyme inhibitors, diuretics and a methylprednisolone pulse therapy followed by oral corticosteroids, azathioprine, colchicine, anticoagulants and methotrexate (replacing the colchicine at hospital discharge for better convenience of administration). After such treatment was begun, the patient had significant improvement. The patient was discharged from the hospital and returned to the outpatient clinic twice - the first 15 days and the second 45 days after hospital discharge; in both outpatient consultations, the patient was well.

## Discussion

Budd-Chiari syndrome, or hepatic venous outflow obstruction, was diagnosed in 30 patients during a 10-year period in a University Hospital in Turkey, and Behçet's disease constituted the major group in the etiologic distribution [12]. Although Budd-Chiari syndrome caused by occlusion of the major hepatic veins, the adjacent inferior vena cava or both, is a rare and serious complication of Behçet's disease, these authors from a University Hospital in Turkey presented 30 cases of Budd-Chiari syndrome, of whom patients with Behçet's disease comprised the largest group; inferior vena cava involvement was more common in these patients [12]. Similar to the literature, the diagnosis of Budd-Chiari syndrome in our patient was also performed with a Doppler ultrasound examination of the suprahepatic and cava veins showing absence of flow in the left suprahepatic vein.

Budd-Chiari syndrome as a complication of Behçet's was seen in four young male patients in another clinical follow-up of the same Turkish University Hospital [13]. Of 220 Tunisian patients who fulfilled the international criteria for diagnosis of Behçet's disease, those with Budd-Chiari syndrome and seven male patients (mean age, 29 years) already diagnosed with Behçet's disease who had Budd-Chiari syndrome were selected [1]. In addition, a young male patient with Behçet's syndrome presenting with Budd-Chiari syndrome who died during an emergency surgery for thrombectomy was reported in another training hospital in Turkey [10]. Despite the gender and origin, our patient with Budd-Chiari syndrome also fulfilled the international criteria for diagnosis of Behçet's disease, and in addition to recurrent oral ulcers, she had recurrent genital ulceration, skin lesions and a positive skin pathergy test.

Evidence in countries where Behçet's disease is prevalent suggests that this disease should be included among the diagnostic possibilities in cases of Budd-Chiari syndrome because the third most common cause from a total of 75 patients diagnosed with Budd-Chiari syndrome was Behçet's disease [14].

Thus, although it has a worldwide distribution, Budd-Chiari syndrome associated with Behçet's disease is more common in the Middle and Far East and affects mainly young men [1,10-13,15].

Although the mortality rate of Behçet's disease is low, most patients with Behçet's disease who develop Budd-Chiari syndrome die as a consequence of hepatic venous outflow obstruction [9,10,13,15]. The hepatic venous outflow obstruction in Behçet's disease is often associated with other venous thrombosis, and the prognosis may be favorable with medical interventions, including anticoagulation, treatment for vasculitis and the use of diuretics when required [11]. Similar to male patients, our patient also had significant improvement in her initial clinical status after treatment.

Despite the prevalence of vascular complications of Budd-Chiari syndrome in patients with Behçet's disease being much greater and more serious in young men, we report a case of Budd-Chiari syndrome in a young woman in association with Behçet's disease. It is possible that the vascular complication in our female patient may be related to her history of alcoholism, which is much more common in men. Although alcoholism is not considered a primary cause of Budd-Chiari syndrome, it is harmful to the liver and could contribute to the onset and a worse outcome of this complication.

## Conclusion

Even though the pathogenic mechanisms are unknown, Budd-Chiari syndrome is a vascular complication that can be associated with Behçet's disease. In countries where the prevalence of Behçet's disease is high, such as Turkey and others in the Middle and Far East, evidence suggests that this disease should be included among the diagnostic possibilities in cases of Budd-Chiari syndrome. Severe vascular complications of Budd-Chiari syndrome in patients with Behçet's disease are much more common in young adult male patients. Although unusual, our patient was a young Afro-Brazilian with Behçet's disease.

## Consent

Written informed consent was obtained from the patient for publication of this case report and accompanying images. A copy of this written consent is available for review by the Editor-in-Chief of this journal.

## Competing interests

The authors declare that they have no competing interests.

## Authors' contributions

All authors read and approved the final manuscript.
